# ATM-independent, high-fidelity nonhomologous end joining predominates in human embryonic stem cells

**DOI:** 10.18632/aging.100197

**Published:** 2010-09-11

**Authors:** Bret R. Adams, Amy J. Hawkins, Lawrence F. Povirk, Kristoffer Valerie

**Affiliations:** ^1^ Departments of Radiation Oncology, Virginia Commonwealth University, Richmond, VA 23298, USA; ^2^ Biochemistry and Molecular Biology, Virginia Commonwealth University, Richmond, VA 23298, USA; ^3^ Pharmacology and Toxicology, Virginia Commonwealth University, Richmond, VA 23298, USA; ^4^ the Massey Cancer Center, Virginia Commonwealth University, Richmond, VA 23298, USA

**Keywords:** BG01V, DSB repair, KU-55933, KU-57788, KU-59436

## Abstract

We recently demonstrated that human embryonic stem cells (hESCs) utilize homologous recombination repair (HRR) as primary means of double-strand break (DSB) repair. We now show that hESCs also use nonhomologous end joining (NHEJ). NHEJ kinetics were several-fold slower in hESCs and neural progenitors (NPs) than in astrocytes derived from hESCs. ATM and DNA-PKcs inhibitors were ineffective or partially effective, respectively, at inhibiting NHEJ in hESCs, whereas progressively more inhibition was seen in NPs and astrocytes. The lack of any major involvement of DNA-PKcs in NHEJ in hESCs was supported by siRNA-mediated DNA-PKcs knockdown. Expression of a truncated XRCC4 decoy or XRCC4 knock-down reduced NHEJ by more than half suggesting that repair is primarily canonical NHEJ. Poly(ADP-ribose) polymerase (PARP) was dispensable for NHEJ suggesting that repair is largely independent of backup NHEJ. Furthermore, as hESCs differentiated a progressive decrease in the accuracy of NHEJ was observed. Altogether, we conclude that NHEJ in hESCs is largely independent of ATM, DNA-PKcs, and PARP but dependent on XRCC4 with repair fidelity several-fold greater than in astrocytes.

## INTRODUCTION

Human embryonic stem cells (hESCs) are notable because they possess the ability to self-renew indefinitely and are capable of differentiating into all tissues of an organism. These cells are able to preserve their genomic and epigenetic integrity to a higher degree than somatic cells [1]. ESCs may use several mechanisms to maintain genomic stability including the up-regulation of DNA repair, the utilization of high-fidelity forms of repair, and the efficient elimination of damaged cells by apoptosis [2-5]. Unrepaired DNA double-strand breaks (DSBs) lead to toxic lesions, chromosomal aberrations and genomic instability that could give rise to cancer [6]. There are two major pathways for DSB repair in mammalian cells; homologous recombination repair (HRR) and non-homologous end joining (NHEJ) [6]. Ataxia telangiectasia mutated (ATM), ATM and Rad3-related (ATR), and DNA-dependent protein kinase catalytic subunit (DNA-PKcs) are members of the PI3K-related kinase (PIKK) family that are activated by DNA damage and are associated with DNA damage check-point signaling and preservation of genomic stability, with all three playing important roles in DSB repair. The form of DSB repair with the highest fidelity is HRR which utilizes homologous sequences from a sister chromatid, homologous chromosome, or repetitive sequence as templates for repairing the damaged DNA.

NHEJ represents the more error-prone form of DSB repair with faster repair kinetics than HRR. Mechanistically this process begins with the binding of the KU70/KU80 heterodimer to the DNA ends which then recruits DNA-PKcs to form the DNA-PK holoenzyme. Before ligation the DNA ends are sometimes resected by the Artemis and/or MRE11/RAD50/NBS1 (MRN) nucleases, followed by XRCC4/DNA Ligase IV/XLF recruitment necessary for resealing [6,7]. Several factors determine whether HRR or NHEJ is employed including stage of the cell cycle, growth factor signaling, and the severity and type of damage [6,8].

Interestingly, a backup NHEJ (B-NHEJ) pathway has been described that utilizes poly (ADP-ribose) polymerase-1 (PARP-1), histone H1, and Ligase III/XRCC1, but not DNA-PKcs as main components for sealing some DSBs [9,10]. Some studies suggest that DNA-PKcs-dependent NHEJ (D-NHEJ) prevents loss of genetic information [11,12], while the less conservative microhomology-mediated end joining (MMEJ) may occur to a greater extent when DNA-PK, and other proteins part of the canonical NHEJ, are absent [13,14]. D-NHEJ reseals DSBs with faster repair kinetics possibly because the repair proteins have greater affinity for the DSB [9,15]. Although the B-NHEJ pathway seems more critically dependent on micro-homology than classical NHEJ, gap-filling of aligned ends has not been observed for B-NHEJ in vitro, and the known NHEJ gap-filling polymerases μ and λ appear to be specifically recruited by the XRCC4/Ligase IV complex [16,17].

PARP-1 binds to DNA at damage sites and catalyses the formation of poly (ADP-ribose) (PAR) on itself and other acceptor proteins including histones [12,18]. PAR formation is believed to alter chromatin structure, protect sites of DNA breaks and attract repair proteins. While knockout of either ATM or PARP-1 individually does not result in lethality, double knockouts are lethal [19]. This suggests that ATM and PARP-1 may act as backup to each other when DNA is faced with harmful DNA breaks. In addition, it was shown that ATM and DNA-PKcs function in the same pathway to ensure cell survival in the absence of PARP-1 [20].

Early in mouse development there is preferential use of HRR compared to NHEJ [21,22]. However, while correlative data suggest that NHEJ may exist in mESC, it is clear that there are differences between the human and mouse systems [23,24], and so far there has been no direct demonstration of NHEJ in hESCs. Recent studies from our laboratory have demonstrated that HRR is utilized extensively by hESCs and that it decreases throughout differentiation to NPs and astrocytes [5]. Importantly, we showed that ATR is imperative for the regulation of DSB repair in hESCs without any apparent involvement of ATM. In support of our findings, it was recently shown that genetic manipulation of hESCs creating an ATM knockout by targeted allele disruption did not lead to significant genetic instability as determined by CGH [25].

Tissue engineering focuses on differentiating stem cells through specific lineages for therapeutic purposes. Preserving genomic stability in these cells is therefore very important and, thus, a better understanding of DNA repair processes occurring in these cells is critical. We report here that rapidly proliferating hESCs utilize NHEJ in a process that is ATM-independent and largely DNA-PKcs-independent and show that upon differentiation of the hESCs to NPs and then astrocytes, the rate of NHEJ progressively increases whereas the fidelity of repair decreases.

## RESULTS

### hESCs utilize NHEJ for DSB repair

**Figure 1.**
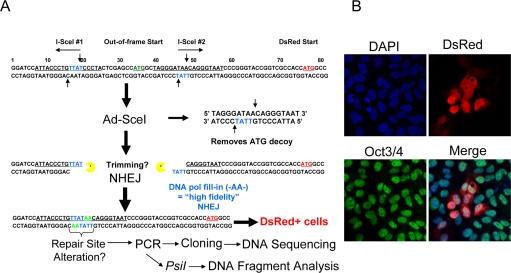
Description of the NHEJ-red repair cassette and processing of I-SceI-digested DNA. (**A**) Schematic of the NHEJ-red cassette. (**B**) Oct3/4 (green) positive hESCs display DsRed (red) 48 h after infection with a multiplicity of infection (MOI) of 30 with Ad-SceI adenovirus. DAPI shows nuclear staining.

Previous work demonstrated that hESCs are highly proliferative cells with strong G2 checkpoints and an absent G1 checkpoint [26]. For this reason it is believed hESCs would depend extensively on HRR. Indeed, we recently showed that hESCs form RAD51 foci, a marker for HRR, far more extensively and express RAD51 at 10-fold higher levels than differentiated astrocytes [5]. We also demonstrated that the relative fast repair kinetics using γ-H2AX foci as surrogate suggested that hESCs have NHEJ [5]. However, both γ-H2AX and 53BP1 foci resolution was relatively unresponsive to a small molecule inhibitor of DNA-PKcs kinase suggesting that NHEJ in hESCs is largely independent of DNA-PKcs [5]. To determine the nature of NHEJ in hESCs in more detail and to clarify the role of DNA-PKcs, we engineered the hESCs with a lentivirus (LV) carrying an I-SceI repair cassette that would make them more amenable to NHEJ analysis (Figure 1A).

BG01V cells were infected with the NHEJ-red LV and stable transductants selected in G418-supplemented medium. A number of clones were isolated and expanded, and one was chosen for further study. The hESC clone was infected with the I-SceI expressing adenovirus and shown to be positive for Oct3/4 nuclear staining, indicating that the cells were hESCs, and positive for DsRed suggesting that they utilize NHEJ. Therefore, the hESCs use NHEJ since the production of DsRed after I-SceI adenoviral infection could only occur by NHEJ (Figure 1B).

### NHEJ detected by genomic DNA qPCR assay

**Figure 2.**
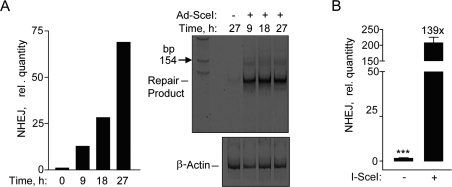
Repair by NHEJ monitored by genomic DNA qPCR. (**A**) Time course exhibiting an increase in SYBR green fluorescence after amplification by qPCR in hESCs (left panel). Polyacrylamide gel showing the NHEJ repair product at ~125 base pair fragment at the indicated times (right panel). (**B**) Relative NHEJ levels after infection with Ad-SceI adenovirus with 30 MOI at 24 h. Fold (x) and statistical significance indicates changes in the relative repair levels when compared to the Ad-SceI infected sample. The difference in increases in the relative quantity of NHEJ at 27 h in (**A**) compared to 24 h in (**B**) is mostly due to a difference in the values obtained from the samples without I-SceI between the two data sets.

After validation of the fluorescence-based NHEJ assay by immunocytochemistry, we utilized a more rapid qPCR assay that also allows for determining NHEJ at earlier time points than by FACS [8]. Genomic DNA qPCR would be a more direct, quantitative method for determining NHEJ that eliminates transcriptional or translational effects that may influence fluorescent protein based DNA repair assays [8]. A time course after Ad-SceI infection showed an increase in NHEJ by SYBR-Green qPCR, and the 125-bp DNA fragment predicted to result from the removal of the 25-bp stuffer from the 150-bp fragment (Figure 2A). Unexpectedly, the 125-bp repair product was amplified much more efficiently than the original uncut sequence, so that the repair product could be detected quantitatively by SYBR-Green qPCR. Using this assay a significant 139-fold increase was detected in the I-SceI infected cells at 24 h compared to uninfected cells (Figure 2B). These results demonstrate that hESCs have the ability to repair DSBs by NHEJ.

### NHEJ levels correlate with differentiation

**Figure 3.**
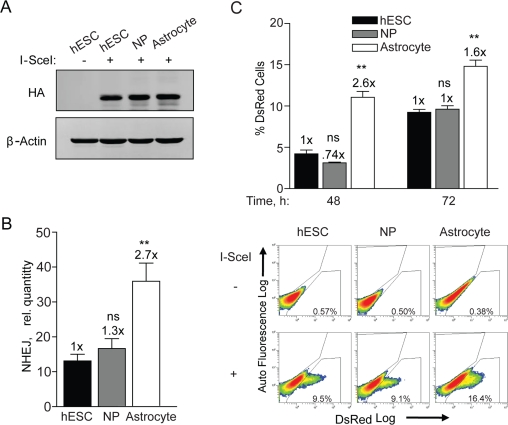
NHEJ occurs with faster kinetics after terminal differentiation. (**A**) hESCs, NPs and astrocytes were seeded and 12 h later infected with Ad-SceI at an MOI of 100. Expression of HA-tagged I-SceI was examined in samples harvested 24 h after infection. (**B**) BG01V/-, NP/-, and astrocyte/NHEJ-red cells were infected with Ad-SceI and collected 24 h later. (Columns) Relative NHEJ levels were determined by genomic DNA qPCR and normalized to β-actin levels; (Error bars) SEM for data sets n = 3. Fold (x) indicates changes in the relative repair levels when compared to the hESC sample. *p < 0.05: **p < 0.01; ***p < 0.001. (**C**) (*Top Panel*) BG01V/-, NP/-, and astrocyte/NHEJ-red cells were infected with Ad-I-SceI at an MOI of 30. DsRed events were determined by FACS 48 and 72 h after infection. Fold (x) and statistical significance indicates changes in the relative repair levels when compared to the hESC sample. (Columns) % DsRed+ cells with 60,000 events collected; (Error bars) SEM for three independent experiments. (*Bottom Panel*) Representative FACS images of DsRed+ cells at 72 h after infection.

Previous work from our group established optimal conditions for the growth and differentiation of hESCs on feeder-free cultures into NPs and astrocytes [5,27,28]. We have not only shown a loss in proliferation after differentiation to astrocytes, but also changes in morphological and phenotypic properties such as increased glutamate uptake associated with astrocytes [27]. Since these cell populations are identical at the genetic level any changes observed are likely due to alterations in epigenetics. Thus, it is possible that adenovirus infection and I-SceI expression may change through differentiation thus accounting for the differences seen in NHEJ. To determine the relative levels of I-SceI expression in these cell populations, hESCs, NPs, and astrocytes were infected with an equal MOI of adenovirus expressing HA-tagged I-SceI. These three cell populations expressed very similar levels of HA-SceI (Figure 3A). This assay would therefore be able to accurately assess any changes in NHEJ repair through differentiation.

In terms of NHEJ, as cells transitioned from hESCs to NPs there was a trend towards increased NHEJ, whereas astrocytes showed a 2.7-fold increase over hESCs when the PCR assay was utilized (Figure 3B). This result was supported by flow cytometry analysis determining the quantity of DsRed positive cells. At 48 h the astrocytes exhibited 2.6-fold more cells expressing DsRed than hESCs and at 72 h there was a 1.6-fold increase (Figure 3C). This result shows that DsRed is produced faster in astrocytes and again that there is no difference between hESCs and NPs. Because of the close fit between the results from the qPCR with that of flow cytometry it is unlikely that astrocytes have a greater ability to express the reporter. Combined, these results show a significant increase in the kinetics of NHEJ upon differentiation of the NPs to astrocytes. Therefore cell cycle stage, cell growth or various multi-potency factors may lead to a differential in the kinetics of, and perhaps also the type of NHEJ repair in these isogenic cell populations.

### ATM and DNA-PKcs kinases are not critical for NHEJ in hESCs

**Figure 4.**
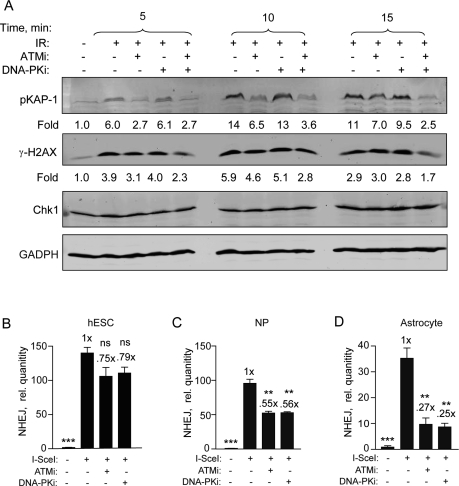
Specific DNA-PKcs and ATM kinase inhibitors become more effective as hESCs differentiate. (**A**) DNA-PKi and ATMi are functioning in hESCs. hESCs were harvested 5, 10, and 15 min after exposure to 6 Gy with or without ATMi (10 μM) and DNA-PKi (2.5 μM) or both. Drugs were added 15 min prior to radiation. Fold change depicts phosphorylation of KAP1 (S824) and H2AX (S139) after normalization to CHK1 (and GAPDH) which served as loading controls. (**B**) BG01V/NHEJ-red (**C**) NP/NHEJ-red and (**D**) astrocyte/NHEJ-red cells were infected with Ad-SceI and then treated with either ATMi at 10 μM or DNA-PKi at 2.5 μM 1 h after infection. Cells were collected at 24 h post-infection. (Columns) Relative NHEJ levels were normalized to β-actin; (Error bars) SEM for data sets n = 3. Fold (x) indicates changes in the relative repair levels when compared to the hESC sample. Differences in the scale of the separate cell populations (**B-D**) are due to variation in the uninfected sample PCR amplification from 3 separate experiments. Statistical significance of differences in NHEJ with respect to cells expressing I SceI with no inhibitor, are indicated.

We, and another group, showed recently that an ATMi was only partially effective at abrogating DSB repair and DNA damage checkpoint signaling in hESCs [5,26]. In order to first confirm that the KU-55933 (ATMi) and KU-57788 (DNA-PKi) small molecule inhibitors were entering the cells, the effect on radiation-induced H2AX (S139) and KAP1 (S824) phosphorylation was examined. KAP1 is involved in chromatin remodeling after DNA damage and its activation is dependent on ATM and DNA-PKcs at early time points [29]. Furthermore, we showed recently that H2AX phosphorylation is completely blocked at early times (≤ 15 min) after irradiation when both drugs are applied to glioma cells [30]. Here, we show that after irradiation p-KAP1 and γ-H2AX are reduced to near basal levels in a time-dependent manner when treated with a combination of ATMi and DNA-PKi (Figure 4A). Therefore, we conclude that both drugs enter hESCs and inhibit the DDR similar to what is seen with glioma cells [30].

We then determined the effect of these drugs on NHEJ using qPCR and show, in line with our previous results [5], that both ATMi and DNA-PKi were relatively ineffective at inhibiting NHEJ repair in hESCs with only 20-25% reduction observed (Figure 4B). However, there was significant inhibition of 40-50% in the presence of either one of these drugs in NPs (Figure 4C). This inhibition becomes even more pronounced when these cells were further differentiated into astrocytes. Here, NHEJ levels were reduced to 25% when cells were treated with the ATMi compared to untreated hESCs and to 27% when treated with the DNA-PKi (Figure 4D). Altogether, these results suggest that NHEJ in hESCs is to a large extent independent on either ATM or DNA-PKcs. However, as hESCs differentiate to NPs and astrocytes, respectively, this dependency progressively increases.

**Figure 5.**
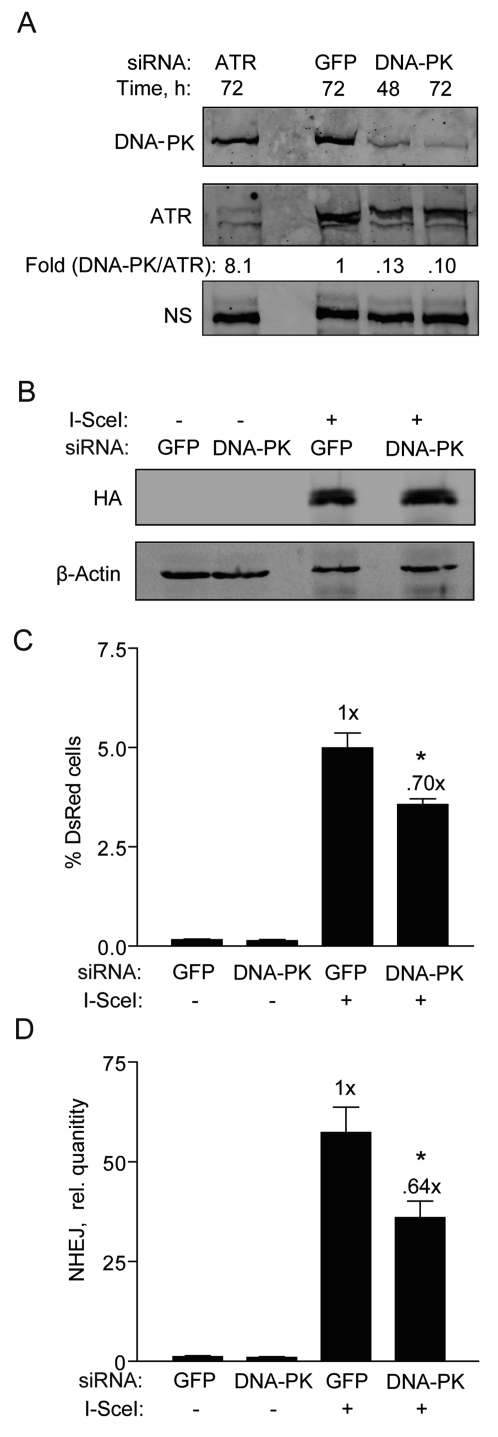
DNA-PKcs knockdown partially reduces NHEJ in hESCs. (**A**) Western blot showing DNA-PKcs expression 48 and 72 h after transfection of BG01V cells with GFP control siRNAs or siRNAs targeting DNA-PKcs or ATR [5]. The fold change in DNA-PKcs was calculated after normalization to ATR which served as a loading control together with a non-specific (N.S.) band. (**B**) Western blot showing HA-SceI levels in BG01V cells 48 h after infection which occurred 48 h after knockdown. (**C**) BG01V/NHEJ-red cells were infected with Ad-I-SceI at 30 MOI, 48 h after knockdown. DsRed events were determined by FACS 48 h after infection. (Columns) % DsRed+ cells with 10,000 events collected; (Error bars) SEM for data sets n = 3. (**D**) BG01V/NHEJ-red cells were infected with Ad-I-SceI at an MOI of 30 48 h after knockdown. Cells were collected at 24 h post-infection. (Columns) Relative NHEJ levels were determined by genomic DNA qPCR and normalized to β-actin levels; (Error bars) SEM for three samples. Fold (x) and statistical significance indicates changes in the relative repair levels compared to the siGFP sample.

To more thoroughly investigate the relative ineffectiveness of the DNA-PKi on NHEJ in hESCs and to better understand DNA-PKcs' role, we transfected the hESCs with siRNA targeting DNA-PKcs and then examined the impact on NHEJ. hESCs transfected with DNA-PKcs siRNAs showed 90% knockdown of DNA-PKcs levels at both 48 and 72 h (Figure 5A). It was also important to analyze the effect of this knockdown on adenoviral expression of I-SceI. DNA-PKcs knockdown did not affect the expression of HA-SceI (Figure 5B). However, only a reduction in NHEJ repair by ~30% was noted (Figure 5C), fully supporting the result with the DNA-PKi. A similar result was obtained with the qPCR assay at an earlier time point of 24 h (Figure 5D). Therefore, in hESCs DNA-PKcs appears to play only a minor role in NHEJ.

### Interfering with XRCC4 function impairs NHEJ

**Figure 6.**
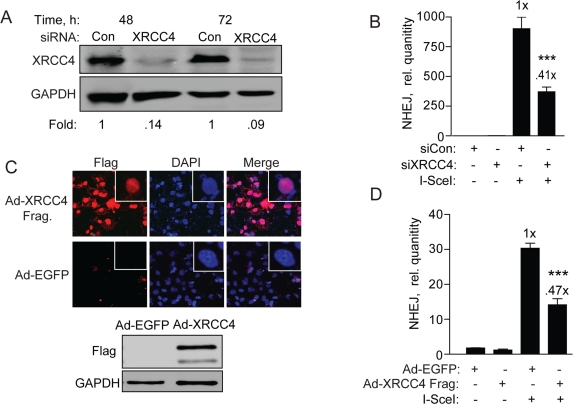
XRCC4 knockdown and expression of a XRRC4 decoy partially reduces NHEJ in hESCs (**A**) XRCC4 knockdown and NHEJ in hESCs. Western blot analysis of extracts with XRCC4 antibody was carried out 48 and 72 h after transfection of BG01V/NHEJ-red cells with non-targeted control siRNAs or siRNAs targeting XRCC4. The fold change in XRCC4 levels was calculated after normalization to GAPDH which served as a loading control. (**B**) BG01V/NHEJ-red cells were infected with Ad-I-SceI at 30 MOI, 48 h after knockdown. Cells were collected at 24 h post-infection for genomic DNA qPCR to determine repair. (**C**) XRCC4 decoy reduces NHEJ in hESCs. Immunocytochemistry (*top panel*) and western blot (*bottom panel*) of BG01V/NHEJ-red cells 48 h after infection with the Ad-Flag-XRCC4_115-293_ virus described previously [31], or an EGFP expressing adenovirus. (**D**) BG01V/NHEJ-red cells were infected with either adenovirus for 48 h and then infected with Ad-SceI and harvested 24 h later. (Columns) Relative NHEJ levels were determined by qPCR and normalized to β-actin levels (Error bars) SEM of three samples. Fold (x) and statistical significance indicate changes in the relative repair levels as compared to those in the I-SceI-expressing cells treated with non-targeting control siRNA.

Canonical NHEJ requires XRCC4/Ligase IV/XLF, which acts as a complex in the final ligation step [6]. XRCC4 is uniquely required for NHEJ and has no other known function than to promote gap-filling and resealing of DSBs during NHEJ. To determine whether repair of the NHEJ-red cassette requires XRCC4 we first knocked down XRCC4 by siRNA followed by genomic qPCR repair assay. XRCC4 was reduced >90% compared to cells transfected with control siRNA at both 48 and 72 h, and repair levels to about 40% (Figure 6A and B), suggesting that XRCC4 and canonical NHEJ is the primary type of repair. Then, to verify this result we infected BG01V/NEJ-red cells with Ad-FlagXRCC4_115-292_ or Ad-EGFP (control) and first examined expression of the XRCC4 protein fragment. Ad-FlagXRCC4_115-292_ expresses a decoy XRCC4 expected to inhibit NHEJ [31]. We found nuclear Flag expression in >70% of the infected hESCs, correlating with the presence of a Flag-containing fragment of the correct size in the Ad-XRCC4 sample and not the Ad-EGFP sample (Figure 6C). When NHEJ was examined, we observed a 50-60% reduction in repair in cells expressing truncated XRCC4, expected to interfere with XRCC4/Ligase IV function, compared to cells infected with Ad-EGFP (control) virus (Figure 6D). Thus, all combined, the primary type of NHEJ utilized in repairing the I-SceI DSB in our construct depends largely on XRCC4 and consequently on classical NHEJ.

### High-fidelity NHEJ decreases as hESCs differentiate

**Figure 7.**
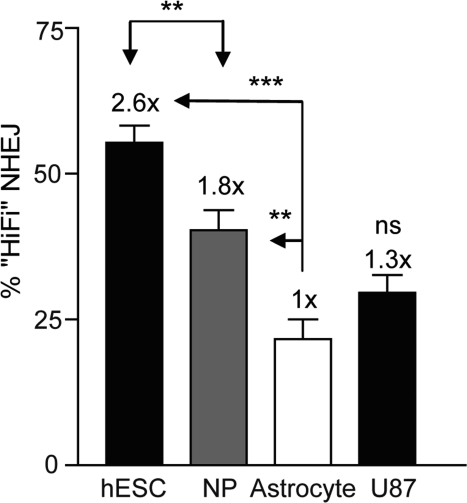
High-fidelity NHEJ decreases through differentiation BG01V/-, NP/-, and astrocyte/NHEJ-red cells were infected with Ad-SceI and collected 24 h after infection. DNA was amplified with Amplitaq Gold and was digested with *Psi*I endonuclease where indicated. (Columns) High-fidelity NHEJ levels were determined by the relative level of the digested portion (*Psi*I-sensitive) of the PCR DNA fragment as a fraction of uncut DNA; (Error bars) SEM for three samples. Fold (x) indicates changes in relative repair levels when compared to the astrocyte samples.

If hESCs rely on alternative forms of repair it is possible there would be a difference in the fidelity with which the repair occurs. As hESCs differentiated to NPs and astrocytes there was a progressive decrease in the extent to which the overhangs were filled in with AA nucleotides indicating high-fidelity NHEJ. hESCs displayed a 2.6-fold higher levels of high-fidelity NHEJ compared to astrocytes, whereas NPs displayed a 1.8-fold increase compared to astrocytes (Figure 7 and Figure S1). A U87 glioma cell clone carrying the NHEJ-red vector [8], showed a 1.3-fold higher level of high-fidelity NHEJ (data not shown) compared to *in vitro* derived astrocytes. In order to verify the results that the ability of *Psi*I to digest the 125-bp PCR product corresponds to high-fidelity NHEJ, DNA sequencing of the cloned PCR fragments was performed. DNA sequencing revealed that 50% of the amplified DNA showed the presence of the *Psi*I site (Table 1 and Table S1), which correlates well with the ~55% obtained by *Psi*I digestion (Figure 7). DNA changes included small deletions of 1-3 nucleotides but no larger deletions or any insertions were noted. In line with this finding, cloning and sequencing of PCR fragments from the U87/NHEJ-red cells showed a similar correlation between *Psi*I digestion and DNA sequence analysis (data not shown). Altogether, high-fidelity repair correlates with replicative growth and cell cycle distribution and was close to 3-fold higher in hESCs than in astrocytes and human glioma cells. Furthermore, our data suggest that when presented as an option partially complementary DNA overhangs are repaired without resection.

**Table 1.** Summary of 28 sequenced clones. Sequencing of plasmid clones recovered after repair of the NHEJ- red cassette.

Sequence	N	%	Deletion
HiFi TTATAA	14	50	none
Non-HiFi			
---ATAA	5	17.9	TT
-TATAA	2	7.1	T
TTAT---	2	7.1	AA
TTATA-	2	7.1	A
TTA---A	2	7.1	AT
TTA----	1	3.6	TAA

DNA sequencing reveals the modifications to the repair site after Ad-SceI infection. The sequence obtained at the repair site, the missing nucleotides, as well as the frequency of the type of DNA damage from twenty-eight clones is shown.

### PARP inhibition does not affect NHEJ but induces DSBs in hESCs

**Figure 8.**
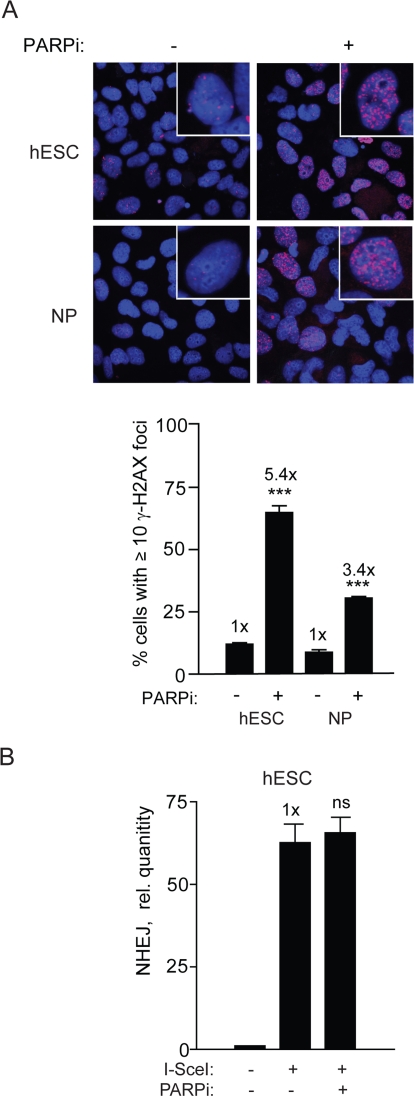
PARPi functions in hESC and induces repair foci but does not affect NHEJ (**A**) PARPi inhibits PARP in hESCs. Images (*top panel*) and graphical depiction (*bottom panel*) of γ-H2AX foci in hESCs and NPs aftercells were treated with PARPi at 3 μM for 16 h. (**B**) PARPi does not affect NHEJ in hESCs. BG01V/NHEJ-red cells were treated with PARPi for 16 h and collected at 24 h after Ad-SceI infection. (Columns) Relative qPCR levels was normalized to β-actin; (Error bars) SEM of three samples. No statistical significance was found between treated and untreated sample.

Our results so far suggests that rapidly dividing hESCs may rely extensively on a DNA-PKcs-independent but otherwise canonical NHEJ. Another type of NHEJ is a microhomology-mediated form of NHEJ (MMEJ) [10], therefore, inhibiting this pathway may reveal the process responsible for NHEJ repair in hESCs. PARP has been shown to be important for MMEJ in mammalian cells [9,10] and therefore a highly specific PARP1/2 inhibitor, KU-54936 (PARPi) could be used to interrogate the possibility that MMEJ is important in hESCs [9,10,32]. In line with current knowledge that PARP inhibition induces DSBs in cancer cells, we show that the PARPi does so also in hESCs and NPs leading to increased γ-H2AX foci formation (Figure 8A). Despite the demonstration that the drug is active in these cells there was no effect on NHEJ (Figure 8B). Thus, PARP-1/2 does not seem to influence NHEJ in hESCs suggesting that B-NHEJ is not critical for repair in our system.

## DISCUSSION

Genomic instability has long been an important issue in cancer and cancer therapeutics but is now also becoming a focus for regenerative medicine therapies. Human embryonic stem cells propagated in culture develop aneuploidy and other DNA rearrangements over time that makes them unsuitable for clinical application. Thus, it is important to understand the mechanisms important for maintaining genomic stability in these cells and in the resulting descendants. This report is beginning to define the role of NHEJ in hESC and in neural cells as a model for studying the DDR and DSB repair in cells of normal human brain.

Current understanding is that the spontaneous mutation rate is lower in embryonic stem cells compared to somatic cells suggesting that these cells have high capacity to repair DNA or that damaged cells easily die [1,4]. There is a requirement for high-fidelity DNA repair in hESCs since they eventually would give rise to an entire organism. Gene knockout studies have demonstrated that mESC cells preferably utilize HRR rather than NHEJ as a principal mechanism of repair and we have demonstrated this holds true also in hESCs [5,21]. It is well established that hESCs predominantly exist in the S/G2 phases of the cell cycle and have a shortened G1 phase where NHEJ dominates in somatic cells [26,33]. Herein, we provide direct evidence that Oct3/4+ hESCs utilize NHEJ as a DSB repair mechanism. While hESCs employ the NHEJ machinery it appears to take a backup role to HRR [5], and the factors and features of this type of repair change through differentiation. Recent findings have suggested that there may be more than one type of NHEJ, with the predominant pathway utilizing DNA-PKcs, and a backup NHEJ pathway utilizing histone H1, PARP-1, and XRCC1/Ligase III [9,10]. However, repair in hESCs seems to primarily use DNA-PKcs-independent NHEJ to support high-fidelity repair without any PARP involvement. Thus, B-NHEJ does not seem to play a major role in the repair of our cassette, however, due to the incomplete elimination of NHEJ when available XRCC4 was knocked down, we cannot rule out that B-NHEJ is serving a back-up role which would only reveal itself when XRCC4/Ligase IV activity is completely eliminated.

It is important to point out a clear distinction between our repair system and other systems examining MMEJ in that we have examined NHEJ when DNA resection is not required and the partial DNA homology resides in the I-SceI overhangs. Other studies have utilized substrates for which MMEJ would require resection to expose the micro-homology to facilitate ligation [34,35]. Most, if not all, of the repair products we identified were small deletions of 1-3 nucleotides and contained a very high level of fidelity. Thus, our study does not address what resection process is functioning in hESCs and neural descendant but rather suggests that when a DNA micro-homology is already present in the DNA overhangs, it can be efficiently used for gap filling and ligation without the need for prior resection.

In examining the kinetics of repair and effects of cell cycle it was shown previously that B-NHEJ has slower repair kinetics and occurs more often in the S/G2 phase of the cell cycle with increased growth signaling greatly enhancing the use of B-NHEJ [36,37]. Herein, we have provided evidence for an increased dependence on D-NHEJ in astrocytes compared to the rapidly proliferating hESCs. Previously, we examined γ-H2AX foci formation and their resolution after irradiation as a surrogate for DSB repair. We showed that as cells advance from the embryonic to more differentiated states DSB repair occurred with faster kinetics and a dependence on ATR and HRR to a dependence on ATM for NHEJ [5]. While the irradiation-induced foci assay is an indirect method and surrogate for repair, in this report we provide direct evidence using an integrated repair cassette that NHEJ occurs with faster kinetics in non-dividing astrocytes than in hESCs and NPs in line with our previous report.

Additionally, we show the ATM and DNA-PK inhibitors have little effect on NHEJ in hESCs but reduce repair in NPs, and even more so in astrocytes. It is possible that these kinase inhibitors may be ineffective since the B-NHEJ pathway may not utilize DNA-PK [10]. However, knockdown of DNA-PKcs in hESCs yielded similar results, suggesting that DNA-PKcs may be helpful but not essential for repair. Although utilization of a B-NHEJ pathway cannot be excluded, the marked decrease in NHEJ seen in XRCC4-knockdown cells suggests that much of the high-fidelity repair seen in hESCs is carried out by the classical NHEJ pathway, even though it is DNA-PKcs-independent. This result is in agreement with previous studies using DSB repair-deficient hamster cells [35], and extracts thereof [38-41], which suggest that KU, XRCC4 and Ligase IV, but not DNA-PKcs, are strictly required for gap filling on aligned DSB ends, a process that is essential for high-fidelity repair of our I-SceI-generated DSB. In support of our findings, a similar conclusion was reached when mouse adipocyte progenitor cells (in which D-NHEJ is not functional) were induced to differentiate, and similarly to what we observe in hESCs, the D-NHEJ repair pathway was only operational after differentiation [42].

It was not surprising that the specific PARPi had no effect on NHEJ in hESC since a similar conclusion was made previously using mESCs [43]. One possible explanation for the lack of an effect of PARPi in hESCs is that these cells are globally euchromatic and have elevated global transcription compared to NPs [44,45]. Therefore, they might not require modification of chromatin for repair, and, interestingly, ATM may only be required for a subset of DSBs associated with heterochromatin [46]. Our experiments only addressed whether PARP is important for resealing NHEJ as a process in the context of a I-SceI-induced break and not at stalled replication forks that might lead to DSBs [47]. However, the PARPi in our study was fully active on hESCs since numerous γ-H2AX foci were seen after exposure to the drug indicating that PARP-1/2 does not affect DNA-PKcs-independent NHEJ in hESCs.

Our previous study showed that hESCs rely extensively on high-fidelity HRR [5]. One possible explanation for the high-fidelity of NHEJ in S or G2 cells is the presence of sister chromatids, which have close physical cohesion that might play an important role in stabilizing the ends of the DSB and preventing degradation or exonuclease activity that leads to deletions [48]. Therefore, this high-fidelity repair may serve as backup should HRR fail and the fidelity may increase from the presence of ATR and other factors only available in S and G2. Alternatively, if NHEJ of the I-SceI break primarily occurs outside of S and G2, for example in the compressed G1 phase, PARP may not serve a critical function.

In summary, NHEJ occurs in hESCs but with slower kinetics than in astrocytes and with a greater extent of high-fidelity repair which is only partially affected by the inhibition of either the ATM or DNA-PKcs kinases. A large fraction of this NHEJ was dependent on XRCC4 and thus would be considered canonical NHEJ. In addition, we were unable to find any involvement of PARP and B-NHEJ in this repair suggesting that NHEJ in hESCs may have unique properties compared to somatic cells.

## METHODS

### Cell culture and treatments

The human ESC line BG01V (ATCC, Rockville, MD) was cultured and differentiated on a feeder free system. BG01V cells are a derivative of BG01 cells with karyotypic abnormalities (49, +12, +17 and XXY) which retain embryonic stem cell markers and characteristics, and the ability to differentiate down a neural lineage [49]. Differentiation was performed to according to published protocols to obtain populations of NPs and astrocytes [5,27,50]. See Supplemental Materials and Methods.

### Antibodies and reagents

Antibodies used were anti-Oct3/4, -Chk1 and -β-actin from Santa Cruz Biotechnology (Santa Cruz, CA), anti-Nestin, -γ-H2AX (clone JBW301), -GFAP, and -Sox2, -Musashi1, -βIII-tubulin, -O1 from Chemicon/Millipore (Billerica, MA), anti-p(S824) KAP1 from Bethyl Laboratories (Montgomery, TX), and anti-HA from Cell Signaling (Danvers, MA). KU-55933 (ATMi), KU-57788 (DNA-PKi), KU-59436 (PARPi) were kindly provided by Mark O'Connor (KuDOS Pharmaceuticals Ltd, Cambridge, United Kingdom) [32,51,52]. All drugs were dissolved in DMSO. *Psi*I was purchased from New England Biolabs (Ipswich, MA).

### Western blotting and immunocytochemistry

Immunocytochemistry and imaging has been described previously [5,53]. Western blotting was performed as described [5,30,53].

### NHEJ repair

BG01V/NHEJ-red cells were isolated by infection of BG01V cells with a lentivirus (WPXLd-2xISceI-DsRed-IRES-NEO) harboring a repair cassette (hereafter referred to as NHEJ-red) positioned upstream of the DsRed reporter gene that was recently described (see Supplemental Methods in [8]), with the exception that an IRES-NEO selection cassette was added (Figure 1A). Cells resistant to G418 were cloned by dilution and screened for the integration of NHEJ-red by infection with adenovirus expressing the I-SceI endonuclease from *Saccharomyces cerevisiae*(Ad-SceI) [8], followed by subsequent analysis of DsRed expression. The NHEJ-red assay is build on a repair cassette having two I-SceI recognition sequences flanking an ATG codon that acts as a decoy preventing translation of the DsRed reporter. Upon cleavage with I-SceI the decoy codon is excised within a 25-bp stuffer fragment. If NHEJ takes place the DNA is sealed and DsRed is expressed from a downstream previously out-of-frame ATG codon (see Figure 1A).

To induce cleavage of NHEJ-red cassette, Ad-SceI (30-100 MOI) was added to the culture medium and cells incubated with virus while slowly rocking for 6 h at 37°C, and then cells were collected at indicated time points for repair analysis. The two I-SceI recognition sites are in opposite orientations, such that when both are cleaved with I-SceI and the stuffer fragment of the vector is excised, two partially complementary 3′ overhangs are generated: -TTAT (5′ → 3′) and TATT- (3′ → 5′). If the generated partially complementary ends anneal without DNA-end resection, a two-base gap will result on both strands which could be filled in by a gap-filling polymerase. This scenario would result in a repair joint with sequence -TTATAA-, which we define as high-fidelity NHEJ. More extensive resection would still result in DsRed expression unless the deletion is so extensive that it removes the downstream DsRed ATG codon or removes the upstream promoter. NHEJ events are then determined by FACS of DsRed positive cells and/or genomic qPCR, or by cloning and DNA sequencing. Flow cytometry was performed on live cells on a Beckman Coulter XL-MC flow cytometer at the Massey Cancer Center Flow Cytometry Facility.

Cells infected with Ad-FlagXRCC4_115-292_ express a truncated version of human XRCC4 that acts as a decoy and presumably interferes with NHEJ resulting in radiosensitization of breast carcinoma cells [31]. BG01V/NHEJ-red cells were infected with either Ad-FlagXRCC4_115-292_ or Ad-EGFP (control) at an estimated MOI of 30 and after 24 h infected or not with Ad-SceI. Twenty-four hours later cells were collected for qPCR repair assay and in a parallel set stained with anti-Flag antibody followed by Alexa-546-conjugated anti-mouse secondary antibody.

### Real-time qPCR assay and *Psi*I digestion

Genomic DNA was extracted using the High Pure PCR Template Preparation Kit (Roche). Amplification of genomic DNA was performed on an ABI 7900HT Real-time qPCR instrument using SYBR Green (ABI, Foster City, CA). Relative NHEJ levels were determined after normalizing to β-actin levels. The PCR primers used for the NHEJ quantification were 5′-CACGAGACTAGCC TCGAGGTTT, 5′-CTTGAAGCGCATGAACTCCTT, and for β-actin were 5′-TCACCCACACTGTGCCCAT CTACGA, and 5′-CAGCGGAACCGCTCATTGCCAA TGG (synthesized by the VCU Massey Cancer Center Nucleic Acids Research Facility). In addition to quantitative SYBR Green PCR, bands were also separated on a 9% non-denaturing polyacrylamide gel, detected by ethidium bromide staining, and imaged on a Typhoon 9410 variable mode scanner (General Electric Healthcare). When amplifying genomic samples to be digested with *Psi*I, Amplitaq Gold Master mix (ABI, Foster City, CA) was used. The samples were then digested with *Psi*I. *Psi*I digestion of the 125-bp PCR fragment generates 77- and 48-bp fragments. Digested bands were separated on a non-denaturing poly-acrylamide gel, stained and imaged as described above. Densitometric values were quantified using the QuantityOne analysis software (Bio-Rad), taking into account the relative size of each fragment.

### DNA sequencing

PCR fragments were cloned and sequenced to determine repair fidelity. DNA was cloned using the TOPO TA Cloning kit (Invitrogen) as described by the manufacturer's instructions and plasmids purified using the 5′ Prime FastPlasmid mini Kit. DNA sequencing of the plasmids with M13 reverse universal primer was performed by the VCU Nucleic Acids Research Facility.

### Knockdown

DNA-PKcs, ATR, and XRCC4 expression was knocked down using the Smartpool siGENOME Cat# M-005030-01-05, M-003202-05, and M-004494-02, respectively. A GFP (5′-GAACGGCAU CAAGGUGAACdTdT-3′), or non-targeting siRNA (D-001210-01-05) was used as a control. All siRNAs were purchased from Dharmacon. hESCs were nucleofected using program A-023 (Lonza Nucleofector II) and Nucleofector Embryonic Stem Cell Kit II solution with 200 nM siRNAs according to the manufacturer's recommendations as described previously [5].

### Statistics

Unpaired two-tailed t-tests were carried out on ≥ triplicate data sets using GraphPad Prism 3.0 (GraphPad Software, Inc.). P-values are indicated as follows: * <0.05; ** <0.01; *** <0.001, ns = not significant. Error bars depict SEM for ≥ triplicate data sets. Statistical significance is marked in all figures comparing data points from different sets at equal time points.

## SUPPLEMENTAL INFORMATION



Figure S1. High-fidelity NHEJ decreases through differentiation. hESCs, NPs, and astrocytes samples were visualized on 9% polyacrylamide gels stained with ethidium bromide. High-fidelity NHEJ was determined by quantification of the PCR amplified DNA resistant and sensitive to *PsiI* digestion (*PsiI*-sensitive) over that of the undigested DNA and the densitometry was adjusted based on the difference in length of each fragment. 125- and 75-bp indicate DNA size markers, and Control + and - indicate unrelated samples infected or not infected with Ad-SceI, respectively.

Table S1. High-fidelity NHEJ Sequencing. DNA sequences of the region flanking the I-SceI DSB in hESCs 24 h after Ad-SceI infection is shown. Twenty-eight clones were sequenced corresponding to Table 1.
