# Supplementary material for: ATM-independent, high-fidelity nonhomologous end joining predominates in human embryonic stem cells
Source: Aging (Albany NY). 2010 Sep 11;2(9):582–96. doi: 10.18632/aging.100197 (PMC2984607; doi:10.18632/aging.100197)
Supplement: Supplementary file 1 [file aging-02-582-s001.doc]

**Supplemental Information**

**Methods**

**Cell culture and treatments.** BG01V cells are embryonic stem cells that are easier to culture than BG01 cells without inadvertently causing cell differentiation. They are a derivative of BG01 cells with karyotypic abnormalities (49, +12, +17 and XXY) which retain embryonic stem cell markers and characteristics, and the ability to differentiate down a neural lineage [49]. Differentiation was performed according to published protocols to obtain populations of NPs and astrocytes [5, 27, 50]. Briefly, BG01V cells were cultured in ES medium as described in [27, 54] consisting of Dulbecco’s modified Eagle’s medium (DMEM)/F12 medium (GIBCO, Grand Island, NY) supplemented with 20% knockout serum replacement (KSR) (GIBCO), 2 mM L-glutamine, 0.1 mM non-essential amino acids, 50 U/ml penicillin, 50 µg/ml streptomycin, 0.1 mM β-mercapto-ethanol, and 5 ng/ml basic fibroblast growth factor (b-FGF) (R & D Systems, Minneapolis, MN). Cells were expanded on dishes of decellularized MEF feeders. Derivation of NPs was performed as described [54]. Briefly, cells were grown on laminin coated dishes in DMEM/F12 medium containing 15% FBS and 5% KSR followed by an additional 7 days in media containing (DMEM/F12, N2 supplement (GIBCO), penicillin/streptomycin, L-glutamine, 5 ng/mL of b-FGF, and 10 ng/mL leukemia inhibitory factor (LIF). The cells were cultured in DN2 media for an additional 7 days to obtain the NP population. NPs were propagated on poly-ornithine- and laminin-coated plates in Neurobasal A Medium (GIBCO) supplemented with B-27 (GIBCO), L-glutamine, and penicillin/streptomycin, 20 ng/mL b-FGF, and 10 ng/mL of LIF. Astrocytes were obtained by exposure of NPs to DMEM and FBS for 25 days on laminin-coated plates as described [27, 50].

Cells were grown on Lab-Tek (Naperville, IL) glass slides. After treatment, cells were fixed with 3% paraformaldehyde, permeabilized with 0.5% Triton-X-100 in phosphate-buffered saline (PBS) and blocked with 10% non-fat dry milk/0.5% goat serum/PBS prior to exposure to primary antibodies. Subsequently, cells were incubated with primary antibodies at dilutions of 1:500 or isotype control non-specific sera (Chemicon) overnight at 4°C in the blocking solution, followed by secondary antibodies Alexa 488 goat anti-rabbit or goat anti-mouse 546 Fab fragment (Invitrogen) at 1:500 dilution, and nuclei counter-stained with DAPI at 1 μg/mL. Cells were imaged and analyzed using a Zeiss LSM 510 Meta imaging system in the Massey Cancer Center Flow Cytometry and Imaging Facility with a 100X objective using the appropriate laser excitation.
