# Supplementary figures and images for: ATM-independent, high-fidelity nonhomologous end joining predominates in human embryonic stem cells
Source: Aging (Albany NY). 2010 Sep 11;2(9):582–96. doi: 10.18632/aging.100197 (PMC2984607; doi:10.18632/aging.100197)

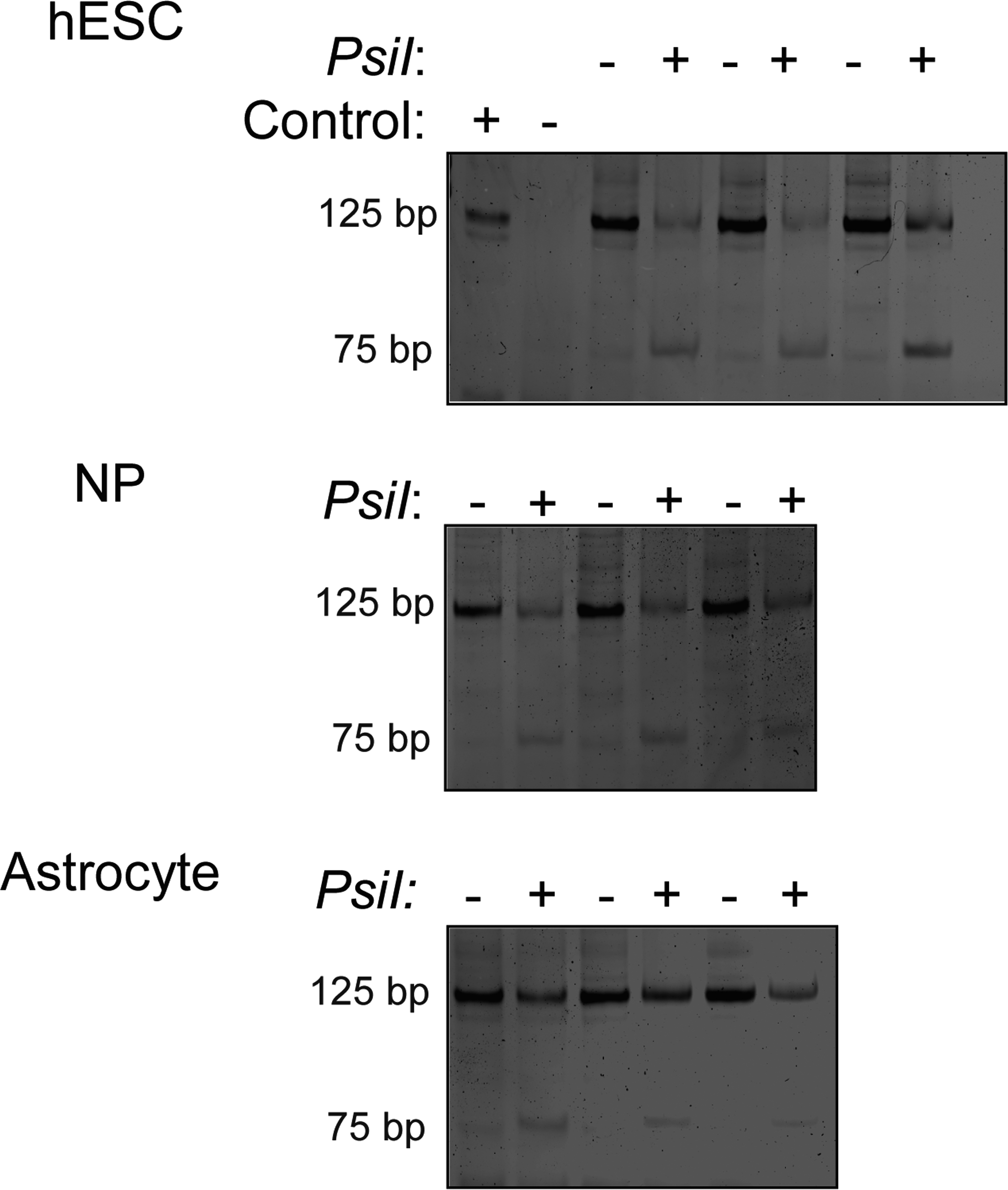

Supplement: Figure S1. — hESCs, NPs, and astrocytes samples were visualized on 9% polyacrylamide gels stained with ethidium bromide. High-fidelity NHEJ was determined by quantification of the PCR amplified DNA resistant and sensitive to PsiI digestion (PsiI-sensitive) over that of the undigested DNA and the densitometry was adjusted based on the difference in length of each fragment. 125- and 75-bp indicate DNA size markers, and Control + and - indicate unrelated samples infected or not infected with Ad-SceI, respectively. [file aging-02-582-s001.tif]

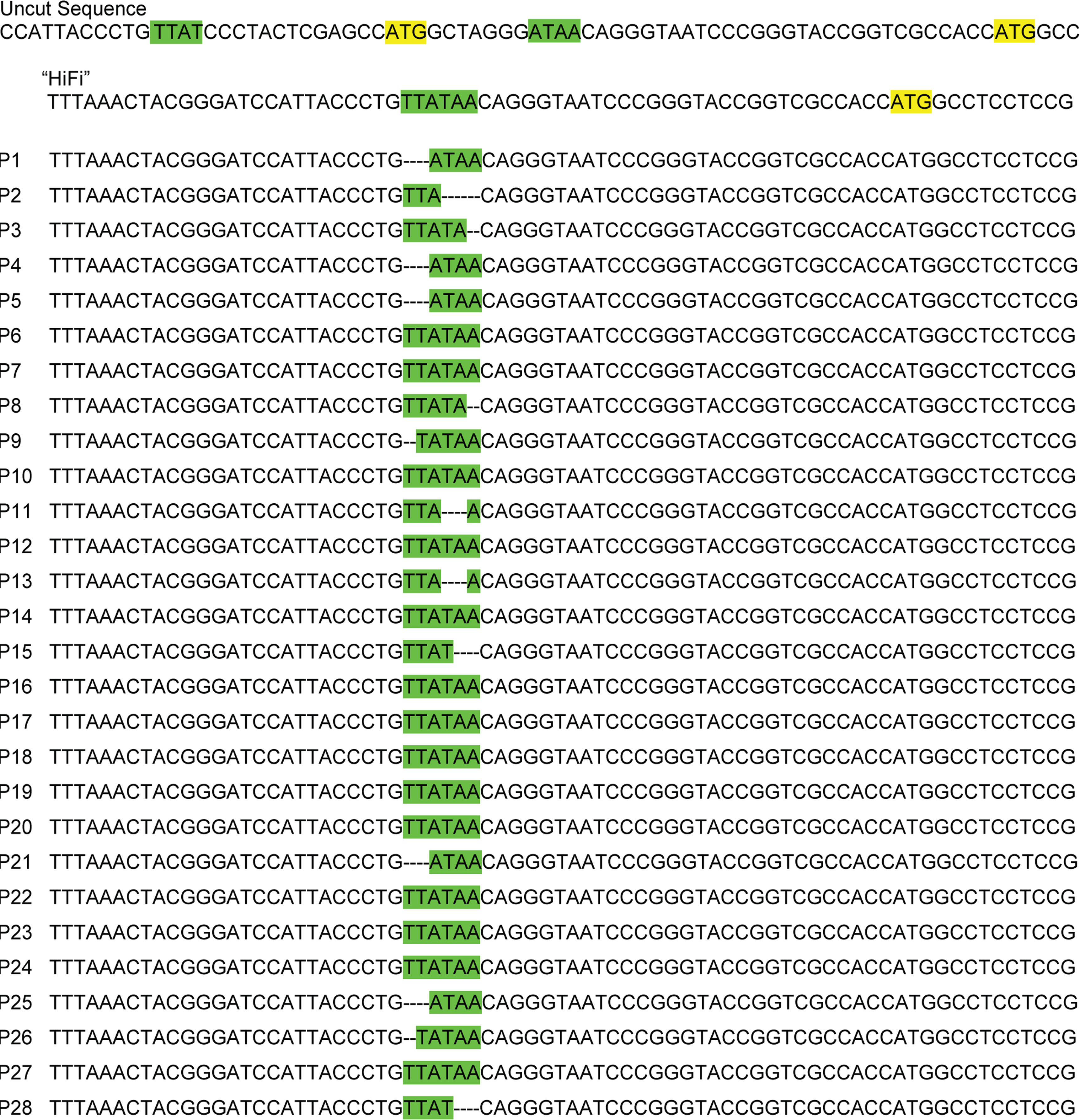

Supplement: Table S1. — DNA sequences of the region flanking the I-SceI DSB in hESCs 24 h after Ad-SceI infection is shown. Twenty-eight clones were sequenced corresponding to Table 1. [file aging-02-582-s002.tif]
